# Crime opportunity theory: the state of the art

**DOI:** 10.1186/s40163-026-00275-z

**Published:** 2026-05-22

**Authors:** Graham Farrell, Nick Tilley

**Affiliations:** 1 School of Law, University of Leeds, Leeds, LS2 9JT UK; 2 Department of Security and Crime Science, University College London, 35 Tavistock Square, London, WC1H 9EZ UK

## Abstract

The 1976 monograph *Crime as Opportunity* initiated a paradigm for how crime and criminality should be understood and controlled. Here we define a crime opportunity as any situation in which the benefits of committing crime outweigh the costs, and group crime opportunity-related theories and concepts under the banner of crime opportunity theory. The study details how reducing crime opportunities has proven successful locally for many different crime types, and how reducing crime opportunities has emerged as the leading explanation for the international crime drop. It concludes that crime opportunity theory should be the principal reference point for explaining and preventing crime and criminality.

## Introduction

The monograph *Crime as Opportunity* (Mayhew et al., 1976), to which this special issue pays tribute on its 50th anniversary, was not well received in some quarters:

“When the contents of *Crime as Opportunity* (Mayhew et al., 1976) were reported in the press in advance of publication an irate psychiatrist wrote to the Home Secretary demanding that he should supress the publication of such manifest nonsense.”

                                                   (Clarke, 1980; 145)

One journal review concluded that:

“Now that the criminological kitchen is becoming so hot, it is as if the [Home Office] Research Unit is looking for a nice, quiet, simple, and nonpolitical corner. It is a touching, if unworldly idea - like playing with one’s toes. But it won’t catch on.”

                                           (Beeson 1976; 20, cited in Clarke & Felson, 2010; 248).

Despite such views the monograph proved robust and initiated the paradigm that evolved into crime science. It comprises a seven-page introductory essay ‘Physical crime prevention: a criminological perspective’, two short case studies on ‘Steering column locks and car theft’ and ‘Damage on buses: the effects of supervision’, and two pages of Concluding Remarks. The introductory essay set the agenda, and the case studies provide supporting evidence. The agenda is that criminology’s obsession with offender dispositions is fruitless, but reducing crime opportunities will prove a practical and efficient means of preventing crime. The case studies are deceptive in appearing mundane to readers unfamiliar with the philosophy they embody, which contrasts with the excitement that they engender among those recognising the broader potential.

The 1976 monograph is written in a clear, efficient, and modest style. Most of Crime Science’s audience will have read the introductory essay to trace the intellectual origins of the journal's subject area, so here we look briefly at the two case studies. The study of London car thefts used a quasi-experimental research design to show that theft of cars with steering column locks compared to other cars was “about three times less in 1973 as it was in 1969.” (p.12). Where previous studies had considered the number of cars on the road as determining opportunity, this study incorporated vehicle security in a central role. It distinguished temporary theft (for joyriding or transportation) from permanent theft (for sale or for parts by more professional thieves), the role of vehicle age and how the volume of ‘stealable’ cars declines only gradually as secure new vehicles replace old ones. It discusses crime displacement and offender adaptations to security (key theft is mentioned on p.13), with a cost-benefit analysis to assess broader utility. We think the case study set a new standard and its influence upon our research relating to the international crime drop and the security hypothesis , discussed later, is clear.

The case study of bus vandalism used a stratified random sample of buses plus observational fieldwork to identify the type and scale of vandalism: holes, seat tears, scratches, and graffiti writing. This was linked to the location on the bus (front or rear, upper or lower deck), the staffing level (single or dual) and bus design (front or rear entrance, and stair location). Principal component analysis gauged vandalism type by location, with analysis of variance to compare between bus types. It found that locations on buses with least surveillance were the most prone to vandalism, with variation by type of vandalism and design of bus, concluding that “the results indicate that situational factors, such as supervision, can play an important part in determining the extent of damage on buses, and indeed, that to reduce bus vandalism these factors must be taken into account.” (p. 28). All this and more in six pages. But it is the approach - the focus on a crime sub-type, combined with the minutiae of context and mechanism – that, for us, embodies the broader lesson. The analytical approach has evolved and has since been applied to organized and transnational crime, terrorist, violence against women and girls, and can be applied to all other types of crime. 

The instigator of *Crime as Opportunity* was Ronald V. Clarke who, in collaboration with Patricia Mayhew, published the British gas suicide story and a study of motorcycle helmets and motorcycle theft in the 1980s. Those studies were named when they were awarded the 2015 Stockholm Prize in Criminology. The gas suicide story showed how seemingly highly-motivated behaviour was dramatically reduced with little displacement when the toxic gas supply used in suicides was replaced with a non-toxic alternative. The motorcycle helmet study (a precursor of which is found on page 18 of *Crime as Opportunity*) found a sharp decline in motorcycle theft in the Federal Republic of Germany. Legislation requiring helmets to be worn meant thieves were conspicuous without a helmet, increasing perceived risk and causing a decline in motorcycle theft. Under Clarke’s leadership, Mayhew and co-author Mike Hough pioneered the British Crime Survey - now the Crime Survey for England & Wales (CSEW) - which became and remains one of the most important sources of information about crime opportunities (Mayhew & Hough, 1992, Hough et al. 2007). As we completed this draft in early 2026, the CSEW was referenced on BBC Radio 4’s *More or Less* programme about statistics in public life: police-recorded violence was causing an outcry because it increased 85% over the last decade in England & Wales, but the CSEW’s more accurate measurements showed that violence had halved! . It turned out that the increase in recorded violence was due to improvements to police recording practices^1^.

In 1980, Clarke apostrophised *‘Situational’ crime prevention: theory and practice* to name the approach outlined in *Crime as Opportunity* (Clarke, 1980). The 1980 study provided the template for his reviews of situational crime prevention (SCP) over the next half century, each introducing new theory and evidence (see e.g. Clarke, 1982, 1995, 2012, Clarke & Homel 1997; Cornish and Clarke 2003, Eck and Clarke 2019). SCP is aligned with other frameworks to reduce crime opportunities, including defensible space (Newman, 1972), Crime Prevention through Environmental Design and environmental criminology (Jeffery, 1971), designing-out crime (Erol et al., 2013), Crime Prevention Through Product Design (Lester, 2001), architecture for crime prevention, Crime Prevention Through Housing Design (Armitage, 2013), problem-oriented policing (Clarke & Goldstein, 2002), the 5 I’s framework (Ekblom, 2011), and the disciplinary approaches detailed in the *Routledge Handbook of Crime Science* (Wortley et al., 2019). Many of these perspectives were nurtured in the *Crime Prevention Studies* series which was the precursor of the *Crime Science* journal and the *Crime Science* book series. In short, *Crime as Opportunity* was the catalyst for SCP and the field now known as crime science.

The rational choice perspective on offender decision-making provided an underlying theory for crime opportunity reduction (Clarke & Cornish, 1985; Cornish & Clarke, 1986). Perfect rationality does not fully represent reality and, consistent with Clarke and Cornish’s interpretation, is complemented by theories of imperfect or bounded rationality. They identified four broad types of decisions: initial involvement (the first offence, often in adolescence); the crime event; continuing involvement in offending; and desistance. Cornish developed the crime event model into crime scripts which has become central to the identification of SCP pinch-points and interventions (Cornish, 1994; Dehghanniri & Borrion, 2019; Leclerc, 2017).

Shortly after *Crime as Opportunity*, the lifestyle theory of victimisation showed how crime opportunities are influenced by variations in everyday movements (Hindelang et al., 1978). The routine activities perspective identified crime opportunities as central to the convergence of potential offenders and suitable targets in the absence of capable guardianship, noting that ‘…the structure of such activities influences criminal opportunity and therefore affects [crime] trends’ (Cohen & Felson 1979; 589) and that the ‘opportunity structure for crime in the United States’ (p.593) was central to the production of its crime rates. These approaches were combined with Lynch’s urban design features of nodes, paths and edges to develop crime pattern theory (Lynch, 1960; Brantingham et al., 2017). Other key developments included the identification of situational precipitators (Wortley, 1997, 1998, 2001, Cornish and Clarke 2003), and further specification of concentrations of crime against victims, places, products, facilities, systems, and offenders (Sidebottom & Tilley, 2017; Tilley, 2024, Chap. 3). John Eck, Shannon Linning and Kate Bowers (2026, in this special issue) identify a broader range of conceptual developments that fall under crime opportunity theory.

## Three definitions 

This section offers three definitions. It defines crime opportunity, proposes use of the term ‘crime opportunity theory’ as a theory for crime science, offers a revised definition of crime science and describes the origins of that term.

### A crime opportunity

The term ‘crime opportunity’ is quite widely used but we have not encountered a satisfactory definition in the academic literature. We suggest:

A crime opportunity is any situation in which the benefits of committing crime outweigh the costs.

The simplicity of this definition belies its general applicability to any crime type and context. A perceived crime opportunity is when the perceived benefits outweigh the perceived costs of committing crime, and p erceptions vary between individuals. Reducing actual or perceived crime opportunities occurs by modifying actual or perceived costs and benefits respectively.

### Crime opportunity theory

The term ‘crime opportunity theory’ is used here to encompass the theories, frameworks and concepts with crime opportunities at their core, that were discussed above. The term is used informally but seldom found in the academic literature, though the plural ‘crime opportunity theories’ is used (such as by Natarajan 2017), with ‘opportunity theory’ or ‘criminal opportunity theory’ appearing elsewhere. We prefer ‘crime’ to ‘criminal’, although we have used the latter on occasion, because of its greater precision. The use of the singular ‘theory’ clarifies that the other theories and frameworks are united by the concept of a crime opportunity. The term highlights the study and prevention of crime opportunities as the central concept of crime science, discussed further below, and so it is a theory for crime science. We suggest this spans all of crime science since engineering, design and other disciplines influence crime through how they change crime opportunities. Overall, the term ‘crime opportunity theory’ allows the relationships between crime science’s other theories and concepts to be more easily understood. It reduces the ambiguity and misunderstanding that can arise with diverse, overlapping and sometimes competing terms in the field. It makes the subject accessible to students, , and provides a clear summary term for others. It is appropriate that it resonates with *Crime as Opportunity*. Note that we also suggest crime opportunity theory applies to criminality, and discuss this further in what follows.

### Crime science

We propose a revised definition of crime science:

Crime science is research and practice to reduce crime opportunities by ethical means using the scientific method and all appropriate disciplines.

This refines Laycock’s definition which refers to crime prevention rather than the reduction of crime opportunities (Laycock, 2005). It has the benefit of identifying the reduction of crime opportunities as central. This acknowledges how crime science is concerned with the design and management of targets, systems, situations and the environment. The definition is intended to include the role of crime precipitators (Wortley, 1997, 1989, 2001) and harm reduction efforts such as the provision of fewer and less harmful weapons which reduce the seriousness of crime opportunities. The ‘all appropriate disciplines’ component of the definition is intended to highlight the role of disciplines outside the social sciences in causing and preventing crime opportunities. Engineering, architecture, design and technology, urban planning, management studies and other disciplines that inform manufacturing and management are important sources of crime opportunities. These disciplines have enormous potential in the reduction of crime opportunities through crime-proofing and security improvements. The scientific method is specified in our definition because, for reasons that are difficult to fathom, some social scientists oppose it or dismiss aspects such as quantitative research. The ‘ethical means’ component highlights that unethical approaches must be actively avoided, and that while democracies typically develop safeguards, this is not always straightforward, and abuse is likely in autocracies (Clarke and Bowers 2017). The definition recognises that studies that use the scientific method and claim crime prevention as their goal are not crime science if they do not reduce crime opportunities. Criminologists focus on offender dispositions, but that is not the realm of crime science, although the evidence discussed below suggests reducing crime opportunities is also the best way to prevent the development of antisocial dispositions, which it does indirectly.

The origins of the term ‘crime science’ lie in the frustration of scholars working on situational crime prevention and environmental criminology, and the tension between their work and how most criminologists focus on offender dispositions. The term ‘environmental criminology’ has been serviceable, but is unsatisfactory because it does not necessarily focus on preventing crime opportunities, and because ‘environmental’ has become an ambiguous term – its use in environmental psychology compared to environmental economics, for example, mean very different things. The term ‘crime science’ as used here was proposed by Ken Pease and adopted by Nick Ross for the Jill Dando Institute of Crime Science at University College London, and implemented by its founding director, Gloria Laycock. The JDI was located in the engineering faculty at UCL at Pease’s suggestion, in line with his proposals for the integration of crime prevention with engineering and other hard science disciplines, including the study of crime futures (Pease 1997, 1998a, b, Association of British Insurers, 2000). Pease studied crime prevention for decades and, along with Ronald V. Clarke and others in the invisible college known as the Environmental Criminology and Crime Analysis group, sought a break with mainstream criminology. The proximal origins of crime science are evident in Pease’s work with the National Board for Crime Prevention where he informed Ross’s thinking (Local Government Chronicle, 1993), and the Foresight Programme (Foresight, 2000) which, as noted by Rose (2004), led to the crime and technology programme introduced by the Engineering and Physical Sciences Research Council to integrate engineering and hard sciences with crime prevention through opportunity reduction^2^.

## Evidence

This section briefly summarizes evidence relating to smaller-scale crime opportunity reduction efforts. It then focuses on evidence relating to the international crime drop as the flagship example of large-scale crime opportunity reduction.

### Small-scale crime opportunity reduction

Many small-scale local studies demonstrate the effectiveness of efforts to reduce crime opportunities. Table 1 is adapted from Clarke’s (2018) review of situational crime prevention. It lists specific crime types and contexts where crime opportunities were successfully reduced.

**Table 1:** Efforts to reduce crime opportunities

Responsible drinking practices in Australia
Street closures to prevent drive-by shootings in Los Angeles
Video cameras in housing for retired persons
Anti-cloning measures for U.S. cell phones
Alley gates to reduce burglaries in Liverpool
Airline baggage and passenger screening
Cash reduction in convenience stores
Anti-robbery screens in London post offices
Automated checking of income by applicants for housing subsidies in Sweden
Systematic cleaning of graffiti on New York City subway cars
Electronic and ink tags on merchandise in clothing stores
Speed cameras and random breath tests
Exact change and drop safes on buses to prevent robbery of bus drivers
Safes with time locks to prevent betting shop robberies in Australia
Removal of gas and electric coin meters from public council housing in England to prevent burglary

These evaluations demonstrate the breadth of crime opportunity reduction efforts. They also demonstrate the specificity, that is, the way in which crime opportunities and thye mechanisms to reduce them vary according to the context. 

### Large-scale: The international crime drop

The international crime drop demonstrates the importance of crime opportunities at scale. High-income countries have experienced decades of declines in many crime types, generally from the 1990s onwards, due to security improvements. Security improvements reduce opportunities for crime which means they are underpinned by crime opportunity theory. We have been unable to identify other explanations that pass the four tests which are the minimum requirement for a valid theory. An explanation for the international crime drop should.

1. account for its occurrence in different countries,
2. account for the previous (post-World War II) long-term increases in crime,
3. account for why some crime types increased over the same period such as theft of phones, and some forms of fraud, and.
4. account for the variation between countries and crime types.

**Fig. 1:**
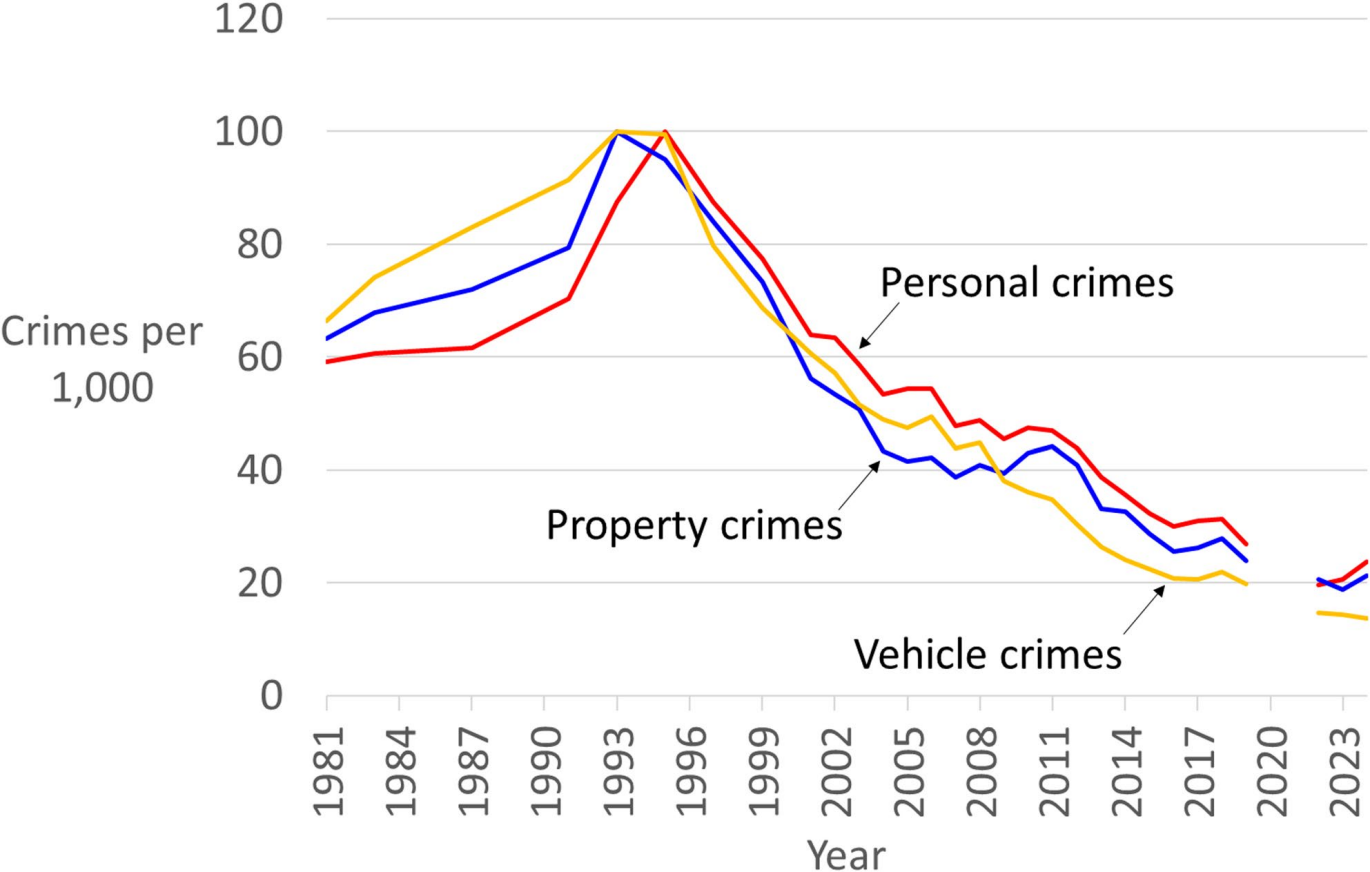
Crime trends in England & Wales indexed to 100 at peak year (*Source*: Crime survey for England and Wales, ONS 2025)

Trends from the Crime Survey for England & Wales since the 1980s are shown in Fig. 1.^3^ Upticks in crime are also consistent with changes to crime opportunities, such as increases due to new frequently stolen goods or offender adaptations to overcome a security device. The outcomes of the four tests applied to a selection of explanations that were offered to explain the international crime drop are shown in Table 2.

**Table 2:** Four tests for a theory of the international crime drop

	Cross-national similarities	Prior crime increase	Other crime increases	Differences within and between countries
Crime opportunity theory (security hypothesis)	✔	✔	✔	✔
Demographics	✔	**X**	**X**	**X**
Imprisonment	**X**	**X**	**X**	**X**
Policing	**X**	✔	**X**	**X**
Illicit drug markets	**X**	✔	**X**	**X**
The internet	✔	**X**	**X**	**X**
Lead poisoning	✔	✔	**X**	**X**
Inflation	✔	**X**	**X**	**X**

The crime drop occurred first in the United States, and researchers in that country pioneered most of the early explanations that were offered. However, the international nature of the crime drop was not widely recognised at the time, so some explanations that initially seemed plausible now appear parochial: they were invalidated because they did not apply to other countries (and have subsequently been invalidated for the US). This included propositions that gun control, the death penalty, increased abortions, large increases in imprisonment, or changes to policing were responsible. These explanations could not explain the crime drop in neighbouring Canada, Western Europe or elsewhere, as one of the American researchers noted:

Closer inspection showed that the timing of the Canadian decline (1991–2000) fit perfectly with the timing of the declining in the United States … The extraordinary similarity of these trends in breadth, magnitude, and timing suggested that whatever was driving the decline in the United States was also operating in Canada. … But … Canada in the 1990s didn’t increase its imprisonment, didn’t hire more police per 100,000 population, and didn’t have anything close to the economic boom we enjoyed south of the border.

                                                  (Zimring, 2006; 619)

Some proposed explanations were incompatible with the long-term increase in crime that occurred in the period after the second world war: imprisonment, for example, had also increased in that period in the US. Changes to policing that were suggested to have a causal role in some cities were found not to have occurred in others where crime also fell, or to have occurred at the wrong time. Proposals that offender numbers or dispositions changed could not account for why some crimes and sub-types, such as phone theft , increased from the 1990s when other crimes were decreasing. However, increased cyber-crime in the 2000s is compatible with crime opportunity theory which explains it as due to the new crime opportunities generated by the internet. This was years after the crime drop began in many countries, so offenders did not switch from analogue to cyber-crimes. More detail on why other hypotheses failed to explain the crime drop are available elsewhere (Farrell, 2013, Farrell et al. 2014, Farrell & Birks, 2018, 2020). While the four tests were proposed in relation to the crime drop, we suggest they can serve as a requirement for an adequate theory of crime and criminality more generally.

### The security hypothesis

The role of security improvements in declining crime was shown through detailed studies of car crime and household burglary (Tilley & Farrell, 2022). Different crimes required different security measures, different security devices had different levels of impact (some being ineffective), and combinations of devices often proved more effective than single ones. Vehicle security improvements were found to have caused all the vehicle theft declines across high-income countries, based on an assessment of studies spanning Australia, Canada, Germany, the Netherlands, the United Kingdom, and the United States (Farrell, 2025). Since all of the drop in vehicle theft was due to security improvements, there is no room left for other explanations.

A review of 50 years of studies of anti-burglary security devices showed how their development and spread explains the decline in US burglaries from 1980 onwards. This squared with studies showing the effectiveness of specific anti-burglary devices (Tseloni et al. 2017a, b; Farrell 2022). 

Similar security improvements occurred across all sectors of society as they were:

“the security measures that hundreds, perhaps thousands, of private and public organizations have been taking in the past 2–3 decades to protect themselves from crime. … This has involved every sector of modern society – shops, banks, schools, colleges, hospitals, offices, transport systems, the airline industry, fast food restaurants, credit card companies, motels, and any other business or organization that is open to some form of criminal exploitation – which is all of them.” (Clarke, 2016; 3).

The international crime drop occurred unevenly. While its benefits were widespread , it occurred more gradually for some than others (Ignatans & Pease, 2016). More affluent households and businesses received benefits first. Then, as with other consumer products, incremental market growth plus competition and economies of scale made good quality security products cheaper and widely available. Subsidised security devices, tax breaks and other interventions might make the spread of suchbenefit more even in the future (Tilley et al., 2011).

### Evaluation science

Evolutionary biologists use fossil evidence to show how and when the evolution of species occurred. Specific geological strata are linked to time periods containing fossils that therefore can be dated to that time period. So, we know when dinosaurs roamed the earth and that this era did not overlap with that of homo sapiens. This means that if ‘human intervention’ was proposed as a hypothesis to explain dinosaur extinction, it would be falsified by the evidence. As with much, perhaps most, scientific knowledge, this was not founded on randomised controlled trials but was based on the triangulation of information from different contexts and times.

The best crime data signatures are like fossil evidence. They serve two functions: to clarify how and why crime change occurred and to rule out alternative explanations (Eck & Madensen, 2009). A more comprehensive listing of indicators is detailed elsewhere (Farrell et al., 2018), but this section details three of the more compelling relating to the role of crime opportunity reduction in the crime drop:

#### Forced and unforced burglaries

**Fig. 2:**
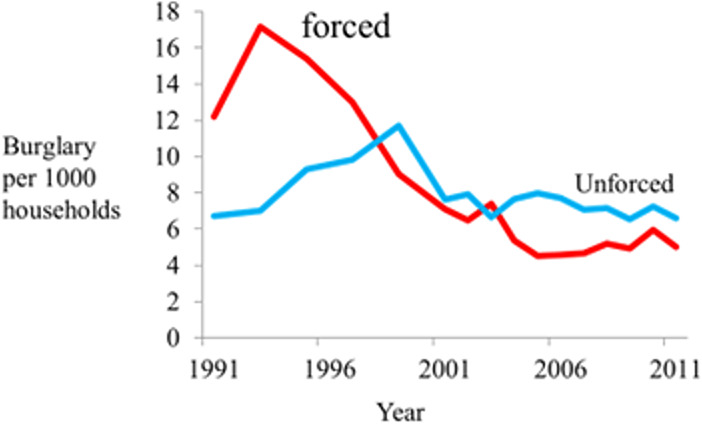
Means of entry for household burglary with entry

There was already clear evidence of a correlation between declining burglary and increased household security devices, with some devices and combinations being more effective. However, the ‘fossil evidence’ was the comparison of the rate of forced and unforced entries to households (Tilley et al., 2015). When the sharp drop in burglary occurred in the early 1990s, it was due to a decline in forced entries via doors and windows (forcing locks, breaking of window and door panes). It was *not* due to a decline in unforced entries: unlocked doors, use of a key, push-pasts, or deception such as pretending to be from a utilities company. The trends are shown in Fig. 2.

The only known explanation consistent with this evidence is that it became more difficult to force doors and windows. That is consistent with them having the improved security that prior research had identified. The continued increase in unforced entries is consistent with burglars using those methods still available to them. The subsequent decline in unforced entries is consistent with young offenders no longer becoming involved because unforced entry requires more search time, skill and experience. This is the fossil evidence that reveals not only how household burglary declined but also rules out other explanations because they do not affect forced rather than unforced entries.

#### Temporary and permanent vehicle theft

**Fig. 3:**
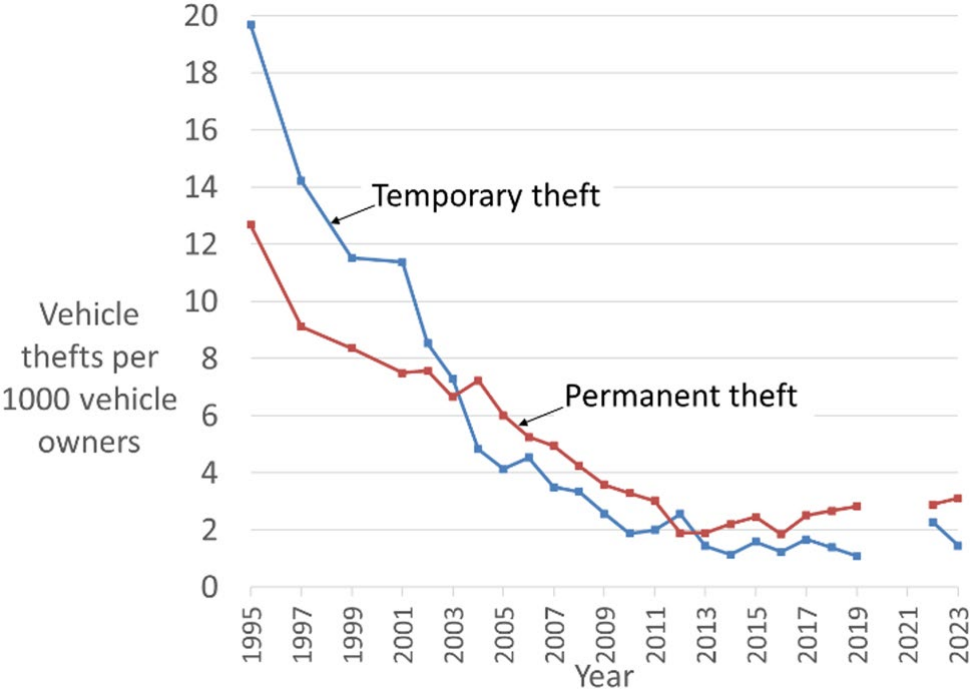
Temporary and permanent vehicle theft in England & Wales (*Source*: Crime survey for England & Wales^4^)

Temporary car theft for joyriding and transportation declined more rapidly than permanent theft for parts, resale or insurance fraud. Temporary theft is mostly by young offenders stealing for joyriding and transportation whereas permanent theft is mostly by older more experienced offenders stealing for profit. The younger offenders were deterred at a higher rate as vehicle theft became more difficult. Importantly though, permanent theft also declined enormously, showing how professional and organised crime can be prevented. The more recent uptick in permanent thefts is likely due to new electronic vulnerabilities in vehicles, that is, offender adaptation to generate a new crime opportunity (Fig. 3). This is fossil evidence because no other proposed explanation for the crime drop can explain these differences.

#### Quasi-experimental evaluation of electronic engine immobilisers

**Fig. 4:**
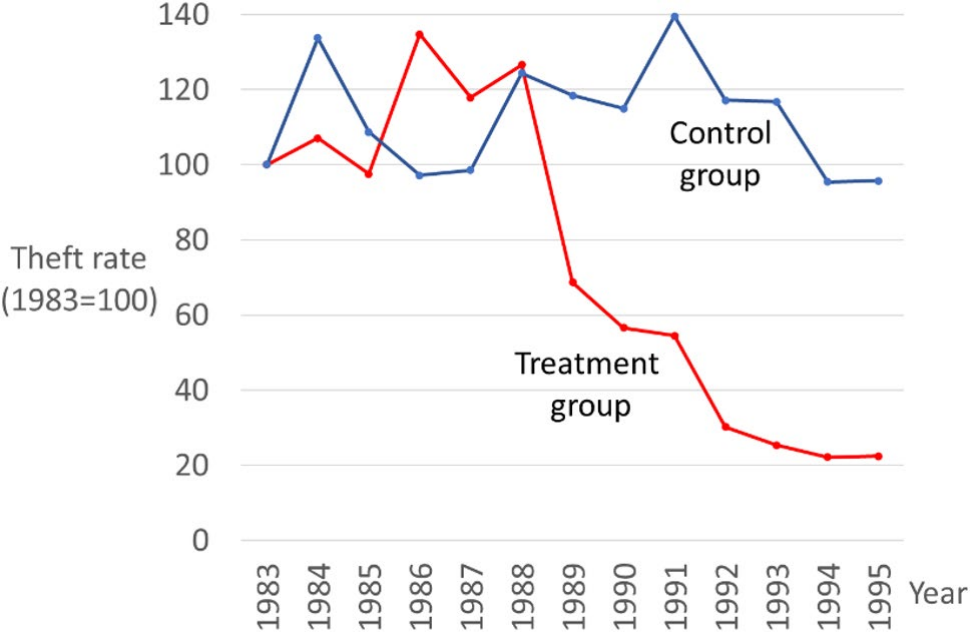
Quasi-experimental evaluation of electronic engine immobilisers in United States (*n* = 141,385 thefts)

A more traditional quasi-experimental evaluation showed the effectiveness of electronic engine immobilisers in the United States. A large treatment group of vehicles received factory-installed electronic engine immobilizers. Their theft rate was compared to a matched control group of vehicles without immobilizers. The cliff-edge 80% drop in theft among the treatment group, shown in Fig. 4, is unambiguous. It squares with a wide range of other evidence and experience in other countries (Farrell, 2025). It is fossil evidence not only because it is clear and unambiguous but also because no other proposed crime drop hypothesis can explain why only the vehicles with electronic immobilisers experienced the theft decline.

### The violent crime drop

The violent crime drop was the result of a domino effect, or diffusion of benefits of, the property crime decline (Farrell et al., 2018). Bank and post office robberies, and robberies of other commercial premises, are acquisitive violent crimes directly prevented by security improvements (though important research to show this remains, to our knowledge, to be undertaken). Bank security improvements including timed vaults, teller screens, marked cash, and other security measures offer a plausible explanation for why bank robberies are much rarer in the 21st century. Violence also declined because of indirect mechanisms, summarised here.

**Fig. 5:**
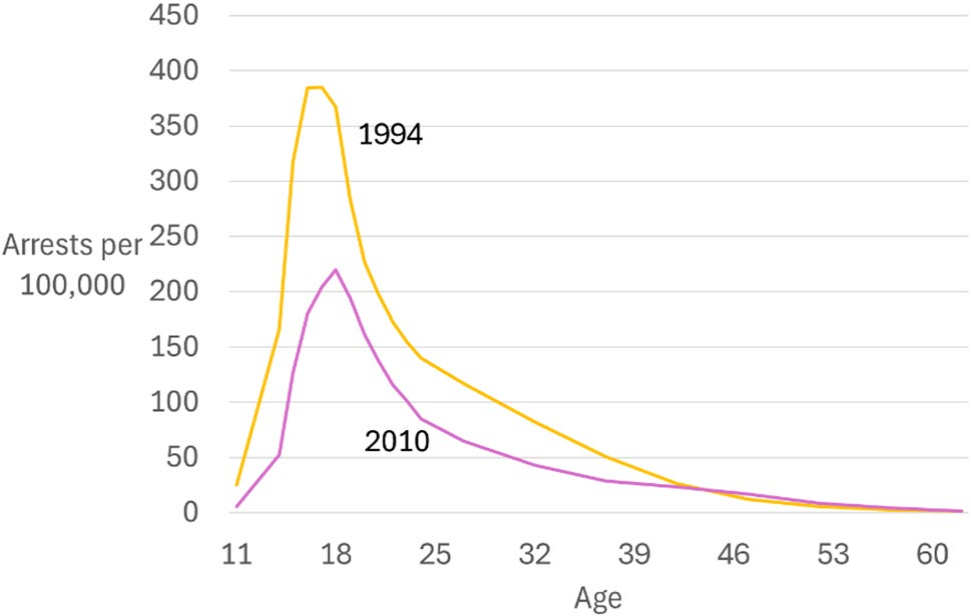
Robbery arrest rate age-crime curves, United States (*Source*: Bureau of Justice Statistics)

Debut crimes are the ‘easy’ property crimes that are often the first offenses committed by youths. As property crimes declined, fewer young people became involved in offending. This meant fewer continued to become habitual offenders. As a result, there was a steep decline in offending in younger age groups (Matthews & Minton, 2017; Farrell et al., 2015). Figure 5 shows robbery arrest rates by age, comparing the high crime year 1994 and the lower crime year 2010. The decline was mostly in juvenile offending. Age-period-cohort analysis showed that whether car theft was easy (before the 1990s) or increasingly difficult (after) determined the cohort offending rate (Dixon & Farrell, 2020). Since young people were no longer tempted to become involved in ‘easy’ property crimes they did not progress to committing violent crime, which declined as a result.

An example where further important research remains to be undertaken is domestic violence. At the time of writing, it had declined for 30 years from the mid-1990s in England & Wales (Walby et al., 2016, ONS 2024). The likely explanation is that domestic violence offenders were more likely to have a history of other forms of offending, and the prevention of those debut property crimes interrupted progression to domestic violence (Dowling et al., 2021).

Property crime is more prevalent than violent crime. Many violent crimes occur as a result of property crimes by two main routes. Violence may co-occur while a property crime is being committed or as a consequence of a property crime. For example, during a household burglary the owner may disturb the offender who responds violently, or a stolen vehicle may be involved in a fatal collision or used to facilitate other crimes. This produces a multiplier effect (Felson & Clarke, 1998; Felson, 2010). As property crime declined it interrupted these mechanisms, preventing violence. This is known as the keystone crime hypothesis because of the similarity with how the removal of the keystone from an arch leads to its collapse. There is also further important research to be undertaken in this area but there is already reasonable evidence that violence declined as a consequence of the property crime decline.

## Discussion and conclusion

This study made three original conceptual contributions: (a) it defined a crime opportunity; (b) it proposed use of the term ‘crime opportunity theory’ to include theories and concepts relating to crime opportunities, and (c) it proposed a new definition of crime science with the prevention of crime opportunities at its core. These proposals are, we suggest, consistent with the spirit and intent of the 1976 monograph *Crime as Opportunity*.

We argued that crime opportunity theory can be used to inform the prevention of all crime types, and has emerged as the leading explanation for ‘today’s foremost criminological phenomenon’ (Clarke, 2021) - the international crime drop. Three fossil data signatures were identified that, in the context of all the other supporting evidence on which they build, provide compelling evidence. This evidence is consistent with crime opportunity theory but inconsistent with other proposed explanations. By the time of writing there are dozens of studies of the role of security in the crime drop and they were given only brief coverage here. The crime drop, of 80 or 90% for many crime types in England & Wales, with similar experiences across high income countries, should also be viewed in the context of the wide array of evidence from local evaluations of interventions for diverse crime types. And, while new and revised explanations for the crime drop appear in the literature from time to time, by 2026 we have not seen any with serious evidence or that come close to passing the four tests. We suggest that, overall, this evidence shows crime opportunity theory to be the leading theory of crime and criminality, and that it should be the foundation of crime control strategies.

This study should not be interpreted as suggesting that crime opportunities explain all variations in crime: there are variations around the mean that can reflect local or other issues. However, the evidence from the international crime drop suggests crime opportunities largely determine the mean trend as well as many of the variations around it. Crime opportunity theory also provides a framework for tackling crime futures, and some of the most important crime science is emerging in that area (Johnson, 2024).

### Criminality as opportunity

The international crime drop research shows how crime opportunities cause criminality. When easy and rewarding crime opportunities are plentiful, more young people are tempted to become involved. When initial involvement proves rewarding, this leads to continued involvement in offending. This produces cohorts with higher rates of continuing involvement in crime. Conversely, as happened with the international crime drop, when crime opportunities become scarcer, fewer adolescents become involved, and cohorts with lower rates of continuing involvement emerge (Dixon & Farrell, 2020).

This is a model of how the prevailing level of crime opportunities causes offending, both at the individual level and among cohorts. It is not circumstances at birth year that determine the rate of offending in a cohort but, rather, the prevailing level of crime opportunities in, for the most part, adolescence. 

Many theories of crime or criminality that are taught in universities around the world are, we suggest, of little practical use either in understanding crime or controlling it. Students should ask their professors how such-and-such a theory can explain long-term trends in crime and ask to be shown the evidence. They should follow up by asking which theories explain the major changes in offending cohorts over time, why crime is so concentrated against particular targets, at particular times and in particular places. They should also ask to see the evidence. Publishers should ask the same questions of textbook authors. Many textbook authors need to update their assessment of theory and evidence, and others to start again from scratch.

Crime opportunity theory encompasses the existing range of theories, concepts, frameworks and approaches with crime opportunities at their core. Crime opportunity theory should be used to inform the prevention of crime and criminality. It provides an accessible terminology and unifying framework within which new concepts and practical approaches can continue to develop.

### Conclusion

Five decades of theory and evidence to date have built on the monograph *Crime as Opportunity*. This has, we suggest, led to an emerging paradigm shift in the understanding of crime and criminality and their control. 

Variations in crime opportunities explain crime trends and concentrations. This includes increases as well as decreases: increased crime opportunities explain increased rates of cyber-dependent and cyber-enabled crime, as well as other new technology-related and emerging crimes. Combined with the problem-solving action-research approach there is a clear roadmap to follow for how to prevent them.

We suggest that crime opportunity theory explains most crime trends and variations in crime at local, national and international levels. It offers an unrivalled general framework for understanding crime and criminality, a basis for identifying ways to pre-empt future crime problems, ways to reduce current crime problems, and a foundation for the further development of crime science as a discipline.

## Data Availability

Not applicable.

## Footnotes

1 BBC Radio 4, More or Less, 06 January 2026 at 14 min 10 s, at. https://www.bbc.co.uk/sounds/play/m002phn6?partner=uk.co.bbc&origin=share-mobile. Note that BBC content is limited to those with access.

2 Alasdair Rose of EPSRC (Rose, 2004) acknowledged the Foresight Crime Prevention panel review (Foresight, 2000) as the starting point for the EPSRC crime and technology initiative which linked engineering and crime.

3 Figure 1 uses incidence rates from the appendix tables to ONS (2025). Here, property crimes per 1000 households included domestic burglary and household theft, personal crimes per 1000 people aged 16 and over included violence, robbery, theft from the person and, other theft of personal property, and vehicle-related theft per 1000 vehicle owners included vehicle-related theft and criminal damage to a vehicle. Data before 2001 and for 2023 and 2024 are for calendar years, with financial years for others.

4 The CSEW uses April–March years from 2001 which are here shown for the main year (so, for example, 2023 is the year from April 2023 to March 2024).

## References

[CR4] Armitage, R. (2013). *Crime Prevention Through Housing Design: Policy and Practice*. Palgrave Macmillan: Basingstoke.

[CR1] Association of British Insurers. (2000). Future crime trends in the United Kingdom. ABI General Insurance Research Report No. 7 (written by K. Pease, M. Rogerson & D. Ellingworth). Association of British Insurers.

[CR3] Brantingham, P. J., Brantingham, P. L., & Andresen, M. A. (2017). The geometry of crime and crime pattern theory. In R. Wortley and M. Townsley (Eds.). *Environmental Criminology and Crime Analysis*, 2nd edition. Routledge: Oxon.

[CR5] Clarke, R. V. G. (1980). Situational’ crime prevention: Theory and practice. *British Journal of Criminology*, *20*(2), 136–147.

[CR6] Clarke, R. V. (1982). Situational crime prevention: Its theoretical basis and practical scope. In M. Tonry, & N. Morris (Eds.), *Crime and Justice 4* (pp. 225–255). University of Chicago Press.

[CR7] Clarke, R. V. (1995). Situational crime prevention. In M. Tonry & D. Farrington (Eds.), *Building a Safer Society: Strategic Approaches to Crime Prevention* (pp. 91–150). Crime and Justice 19. Chicago: University of Chicago Press.

[CR8] Clarke, R. V. (2012). Opportunity makes the thief. Really? And so what? *Crime Science*, *1*(2), 1–9.

[CR9] Clarke, R. V. (2016). Criminology and the fundamental attribution error. *Criminologist*, *41*(3), 1–7.

[CR10] Clarke, R. V., & Cornish, D. B. (1985). Modelling offenders’ decisions: A framework for research and policy. In M. Tonry (Ed.), *Crime and Justice: An Annual Review of Research* (Vol. 6, pp. 147–185). University of Chicago Press.

[CR12] Clarke, R. V., & Felson, M. (2010). The origins of the routine activity approach and situational crime prevention. In F. Cullen, C. Jonson, A. Myer, & F. Adler (Eds.), *The Origins of American Criminology*. Transaction. pp. 245-260

[CR11] Clarke, R. V., & Goldstein, H. (2002). Reducing theft at construction sites: Lessons from a problem-oriented project. In N. Tilley (Ed.), *Analysis for crime prevention* (pp. 89–130). Crime Prevention Studies 13. Monsey, NY: Criminal Justice Press.

[CR66] Clarke, R. V. (2021). Regulating crime and the international crime drop. *International Criminal Justice Review*, *31*(3), 257–259.

[CR67] Clarke, R. V., & Bowers, K. (2017). ) Seven misconceptions of situational crime prevention. In: Tilley, N. and Sidebottom, A. (Eds.) Handbook of Crime Prevention and Community Safety, 2nd edition. Abingdon: Routledge, 109–142.

[CR71] Clarke, R. V., & Homel, R. (1997). A Revised Classification of Situational Crime Prevention Techniques. In S.P. Lab (Ed.) Crime Prevention at a Crossroads. Cincinnati, OH: Anderson. pp. 17-27.

[CR65] Cohen, L. E., & Felson, M. (1979). Social change and crime rate trends: A routine activity approach. *American Sociological Review*, *44*(4), 588–608.

[CR14] Cornish, D. B. (1994). The procedural analysis of offending and its relevance for situational prevention. *Crime Prevention Studies*, *3*, 151–196.

[CR13] Cornish, D. B., & Clarke, R. V. (Eds.). (1986). *The Reasoning Criminal: Rational choice perspectives on offending*. Springer.

[CR15] Dehghanniri, H., & Borrion, H. (2019). Crime scripting: a systematic review. *European Journal of Criminology*, *18*(4), 504–525. 10.1177/1477370819850943

[CR16] Dixon, A., & Farrell, G. (2020). Age-period-cohort effects for half a century of motor vehicle theft in the United States. *Crime Science*, *9*(11), 1–17. 10.1186/s40163-020-00126-5 PMC7527298 33020727

[CR17] Dowling, C., Boxall, H., & Morgan, A. (2021). *The criminal career trajectories of domestic violence offenders. Trends & Issues in Crime and Justice No. 624*. Australian Institute of Criminology: Canberra.

[CR18] Eck, J. E., & Madensen, T. (2009). Using signatures of opportunity structures to examine mechanisms in crime prevention evaluations. *Crime Prevention Studies*, *24*, 59–84.

[CR19] Ekblom, P. (2011). *Crime Prevention, Security and Community Safety using the 5Is Framework*. Palgrave Macmillan.

[CR20] Erol, R., Press, M., Cooper, R., & Thomas, M. (2013). Designing-out crime: rising awareness of crime reduction in the design industry. *Security Journal*, *15*, 49–61. 10.1057/palgrave.sj.8340104

[CR21] Farrell, G. (2013). Five tests for a theory of the crime drop. *Crime Science*, *2*(5), 1–8.

[CR22] Farrell, G. (2022). Forty years of declining burglary in the United States: Explanation and evidence relating to the security hypothesis. *Security Journal*, *35*(2), 444–462.

[CR23] Farrell, G. (2025). The great American car crime decline. *Security Journal*. 10.1057/s41284-024-00452-2 10.1057/s41284-024-00452-2 PMC12559002 41164263

[CR25] Farrell, G., & Birks, D. (2018). Did cybercrime cause the crime drop? *Crime Science*, *7*(8), 1–4. 30956932 10.1186/s40163-018-0082-8 PMC6428390

[CR24] Farrell, G., & Birks, D. (2020). Further rejection of the cybercrime hypothesis. *Crime Science*, *4*(9), 1–4.

[CR27] Farrell, G., Laycock, G., & Tilley, N. (2015). Debuts and legacies: The crime drop and the role of adolescence-limited and persistent offending. *Crime Science*, *4*(16), 1–10.

[CR28] Farrell, G., Tseloni, A., & Chenevoy, N. (2018). ‘Did violence fall after property crime?’ in G. Farrell and A. Sidebottom (Eds.). (2018). *Realistic Evaluation for Crime Science: Essays in Honour of Nick Tilley*. London: Taylor and Francis.(pp. 141–155).

[CR69] Farrell, G., Tilley, N., & Tseloni, A. (2014). Why the crime drop? In M. Tonry (Ed.), *Why Crime Rates Fall and Why They Don’t, volume 43 of Crime and Justice: A review of research* (pp. 421–490). University of Chicago Press.

[CR29] Felson, M. (2010). What every mathematician should know about modelling crime. *European Journal of Applied Mathematics*, *21*, 275–281.

[CR30] Felson, M., & Clarke, R. V. (1998). *Opportunity makes the thief: Practical theory for crime prevention*. Home Office. Police Research Series paper 98.

[CR31] Foresight. (2000). *Turning the Corner*. UK Foresight Programme.

[CR32] Hindelang, M., Gottfredson, M. R., Garafalo, J. (1978) , Victims of Personal Crime: An Empirical Foundation for a Theory of Personal Victimization. Ballinger:, Cambridge, M. A., *y*.

[CR64] Hough, J. M., & Clarke, R. V. (2007). British Crime Survey after 25 years. In M. Hough, & M. Maxfield (Eds.), *Surveying Crime in the 21st Century*. Criminal Justice.

[CR68] Ignatans, D., & Pease, K. (2016). The global crime drop and changes in the distribution of victimisation. *Crime Science*, *5*(11), 1–6.

[CR33] Jeffery. C. R. (1971). *Crime Prevention Through Environmental Design*. Beverly Hills: Sage.

[CR34] Johnson, S. D. (2024). Identifying and preventing future forms of crime using situational crime prevention. *Security Journal*, *37*, 515–534.

[CR35] Laycock, G. (2005). Defining crime science. In M. Smith, & N. Tilley (Eds.), *Crime Science: New approaches to preventing and detecting crime*. Willan. pp.3-25.

[CR36] Leclerc, B. (2017). Crime scripts. In R. Wortley and M. Townsley (Eds.). *Environmental Criminology and Crime Analysis*, 2nd edition. Routledge: Oxon.

[CR37] Lester, A. (2001). *Crime Prevention Through Product Design, Australian Institute of Criminology Trends & Issues in Crime and Criminal Justice No. 206*. Australian Institute of Criminology.

[CR38] Local Government Chronicle (1993). First meeting of National Board for Crime Prevention, at https://www.lgcplus.com/archive/first-meeting-of-national-board-for-crime-prevention-23-07-1993/, 23 July 1993, accessed 03 September 2025.

[CR39] Lynch, K. (1960). *The Image of the City*. MIT Press.

[CR42] Matthews, B., & Minton, J. (2017). Rethinking one of criminology’s ’brute facts’: The age-crime curve and the crime drop in Scotland. *European Journal of Criminology*. 10.1177/1477370817731706 10.1177/1477370817731706 PMC5952325 29805319

[CR41] Mayhew, P., & Hough, M. (1992). The British crime survey: The first ten years. *International Journal of Market Research*, *34*(1), 1–15.

[CR40] Mayhew, P., Clarke, R. V. G., Sturman, A., & Hough, J. M. (1976). *Crime as Opportunity*. Home Office Research Study No. 34. London: Home Office.

[CR72] Natarajan, M. (2011). *Crime Opportunity Theories: Routine Activity, Rational Choice and their Variants*. Routledge.

[CR43] Newman, O. (1972). *Defensible space. *. New York: Macmillan.

[CR44] Office for National Statistics (ONS) (2024). Domestic abuse prevalence and trends, England and Wales: year ending March 2024. Office for National Statistics Centre for Crime and Justice, (27 November 2024 ).

[CR45] Office for National Statistics (ONS). (2025). *Crime in England and Wales: appendix tables, December 2024 edition*. Office for National Statistics Centre for Crime and Justice.

[CR46] Pease, K. (1997). Predicting the future: The roles of routine activity and rational choice theory. In G. Newman, R. V. Clarke, & S. Shoham (Eds.), *Rational Choice and Situational Crime Prevention: Theoretical foundations*. Dartmouth. pp.233-246.

[CR47] Pease, K. (1998a). Crime, labour, and the wisdom of Solomon. *Policy Studies*, *19*(3/4), 255–265.

[CR48] Pease, K. (1998b). Changing the context of crime prevention. In P. Goldblatt, & C. Lewis (Eds.), *Reducing offending: An assessment of research evidence on ways of dealing with offender behaviour. Home Office Research Study 187*. Home Office. pp.39-48

[CR49] Rose, A. (2004). Think crime! Presented to a conference on How Can Science Support the Home Office in Reducing and Detecting Crime, Improving Security, Controlling Immigration and Managing the Prison Service? The Royal Society, London (available via: https://www.foundation.org.uk/getattachment/3b7979f8-c2c3-4a77-8e84-dbc3f293edca/20040526_rose.pdf accessed 16 July 2025.

[CR50] Sidebottom, A., & Tilley, N. (2017). Designing-out crime in systems: Introducing leaky systems, In A. Sidebottom and N. Tilley (Eds.) *Handbook of Crime Prevention and Community Safety*, 2nd edition.

[CR51] Tilley, N. (2024). *Better Crime Prevention*. Routledge.

[CR53] Tilley, N., & Farrell, G. (2022). Security and international crime drops. In M. Gill (Ed.). *The Handbook of Security, 3rd Edition*. Basingstoke: Palgrave Macmillan, pp. 891–907.

[CR52] Tilley, N., Tseloni, A., & Farrell, G. (2011). Income disparities of burglary risk: Security availability during the crime drop. *British Journal of Criminology*, *51*, 296–313.

[CR54] Tilley, N., Farrell, G., & Clarke, R. V. (2015). Target suitability and the crime drop. In M. Andresen, & G. Farrell (Eds.), *Routine Activities and the Criminal Act* (pp. 59–76). Palgrave Macmillan.

[CR55] Tseloni, A., Thompson, R., Grove, L., Tilley, N., & Farrell, G. (2017a). The effectiveness of burglary security devices. *Security Journal*, *30*(2), 646–664.

[CR56] Tseloni, A., Farrell, G., Thompson, R., Evans, E., Grove, L. E., & Tilley, N. (2017b). Domestic burglary drop and the security hypothesis. *Crime Science*, *6*(3), 1–16. 10.1186/s40163-017-0064-2 PMC7089640 32226710

[CR57] Walby, S., Towers, J., & Francis, B. (2016). Is violent crime increasing or decreasing? A new methodology to measure repeat attacks making visible the significance of gender and domestic relations. *British Journal of Criminology*, *56*, 1203–1234.

[CR58] Wortley, R. (1997). Reconsidering the role of opportunity in situational crime prevention. In G. Newman, R. V. Clarke, & S. G. Shohan (Eds.), *Rational Choice and Situational Crime Prevention*. Ashgate Publishing. pp.65-82/

[CR59] Wortley, R. (1998). A two-stage model of situational crime prevention. *Studies on Crime and Crime Prevention*, *7*, 173–188.

[CR60] Wortley, R. (2001). A Classification of techniques for controlling situational precipitators of crime. *Security Journal*, *14*, 63–82.

[CR61] Wortley, R., Sidebottom, A., Tilley, N., & Laycock, G. (2019). *Routledge Handbook of Crime Science*. Routledge.

[CR62] Zimring, F. E. (2006). The value and necessity of transnational comparative study: Some preaching from a recent convert. *Criminology*, *5*(4), 615–622.

